# Uranium hydroxide/oxide deposits on uranyl reduction†

**DOI:** 10.1039/d3ra02899b

**Published:** 2023-05-31

**Authors:** Kazuki Ouchi, Daiju Matsumura, Takuya Tsuji, Tohru Kobayashi, Haruyoshi Otobe, Yoshihiro Kitatsuji

**Affiliations:** a Nuclear Science and Engineering Center, Japan Atomic Energy Agency 2-4 Shirakata, Tokai-mura Naka-gun Ibaraki Japan ouchi.kazuki@jaea.go.jp; b Materials Sciences Research Center, Japan Atomic Energy Agency 1-1-1 Koto Sayo Hyogo 679-5148 Japan

## Abstract

We clarified the chemical reaction of deposits following the reduction of uranyl ions (U^VI^O_2_^2+^) from the results of electrochemical quartz crystal microbalance, impedance spectra, and X-ray absorption fine structure measurements. We propose the following deposition mechanism: (1) U^IV^ is formed by the disproportionation of U^V^, (2) U^IV^ forms U^IV^ hydroxide deposits, and (3) finally, the hydroxide deposits change to U^IV^ oxide, which generally have a larger electrical resistance than the hydroxide form.

## Abstract 2

We clarified the chemical reaction of deposits following the electrolytic U^VI^O_2_^2+^ to U^V^O_2_^+^ reduction.

## Graphical abstract

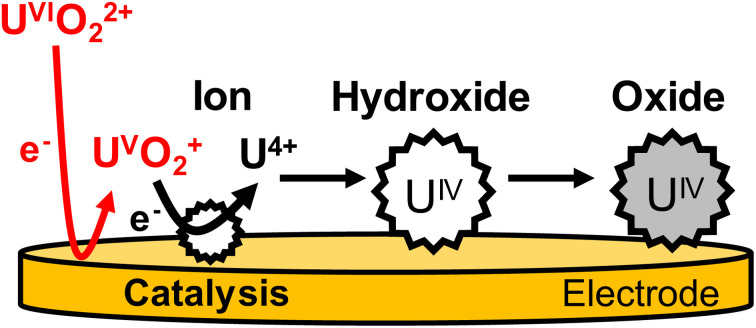

## Introduction

Uranium (U) is one of the actinide (An) elements, which can exist in oxidation states from U^III^ to U^VI^ in aqueous solution.^1,2^ The reactivity of U ions greatly varies depending on the oxidation state (reactivity with an anion: U^IV^ > U^VI^ > U^III^ > U^V^) and U^IV^, which has a large electric charge, easily forms colloid deposits in the form of hydroxide complexes and aggregates in weak acid solutions. It is essential to understand the behavior of U in the environment for the safety evaluation of the geological disposal of high-level radioactive waste. This is because colloids moving in groundwater show complicated behaviors, such as moving in the aquifer and adsorbing into the soil.^3–7^ The retained water generated in a nuclear reactor from the accident at Fukushima Daiichi Nuclear Power Station (referred to as FDiNPS) was analyzed and particulate solids containing alpha nuclides, such as U were reported.^8^ The formation mechanism is not clear. The system in a nuclear reactor is considered to be a non-uniform system and contains U, as shown below. U is thought to have complex reactions, including (1) oxidation state changes in a redox atmosphere and (2) the formation of colloids and particles in the FDiNPS retained water. It is important to understand the valence changes and the formation of colloids/particles to determine how uranium behaves and how it should be properly managed in the treatment process for the retained water in the building and the storage tank of FDiNPS. Therefore, the formation reactions of deposits, as mentioned above, should be clarified.

In solution chemistry studies of An elements, many equilibrium–theoretical parameters have been reported thus far, such as complex formation and solubility constants at each oxidation state.^9–12^ Also, uranium speciation in natural waters and in the environment has been studied by various methods, including anion exchange, laser spectroscopy, X-ray absorption spectroscopy, and Raman spectroscopy.^13–17^ Most of those studies were in equilibrium and do not include dynamic reaction processes. As the deposition of U includes changes in the oxidation state nanoparticulation following reduction, it was reported that the reduction of U^VI^ to U^IV^ by Fe^II^ and green rust leads to the formation of U^IV^O_2_ nanoparticles.^18,19^ Those studies are limited to the speciation of the final product and the nanoparticulation mechanism following reduction has not been described in detail.

We focused on deposition following the electrolytic U^VI^ to U^V^ reduction in a weak acid solution^20,21^ to understand the FDiNPS retained water. We found that electrolytic reduction formed U deposits on the electrode surface using electrochemical quartz crystal microbalance (EQCM) measurements.^21^ Moreover, the deposition process is not direct but proceeds in three stages: (1) the process from the beginning of electrolysis to the beginning of deposition, (2) the growth process of deposits that temporarily shows a fast reduction and deposition rate, and (3) the process in which the deposits continue to react at a constant reduction and deposition rate.^21^ In the second stage, the U deposits enhance the disproportionation and electrolytic reduction rates of U^V^ to U^IV^.^20^ However, the mechanism of U deposition following reduction has not been clarified to date. This study aims to elucidate the deposition reaction following the reduction of the uranyl ion using EQCM, impedance spectra and X-ray absorption fine structure measurements.

## Experimental

### Chemicals

All reagents and solutions were prepared using ultrapure water (18.2 MΩ cm) purified by a Simplicity UV system (Merck Millipore, Burlington, United States). The HClO_4_ solution of U^IV^O_2_^2+^ was prepared as follows. Appropriate amounts of natural uranium metal of chemical purity higher than 99.99% (JAERI-U4, natural isotopic abundance; JAEA) were dissolved in 7 mol dm^−3^ HNO_3_ solution. The HNO_3_ solution of U was heated to near dryness. Then, the residue was dissolved in 4 mol dm^−3^ HClO_4_ and heated to near dryness at the fuming temperature of HClO_4_. The fuming procedure was repeated three times to adjust the U oxidation state to be hexavalent.^22^ The residue obtained was dissolved in 1 mol dm^−3^ HClO_4_ to yield a 0.2 mmol dm^−3^ stock solution. The U^4+^ and U^V^O_2_^+^ solutions were prepared by bulk electrolysis, applying a −0.35 V (*vs.* Ag/AgCl) potential using a ALS730E model analyzer (BAS, Tokyo, Japan) at 0.1 mol dm^−3^ HClO_4_ or pH 2.8 conditions.^12^ The oxidation state of the U ion was confirmed by UV-vis spectroscopy using a V-730 model spectrophotometer (JASCO, Tokyo, Japan).

### Electrochemical measurements

EQCM measurements were carried out with a BAS ALS420C EQCM analyzer (BAS, Tokyo, Japan) using a 7.995 MHz AT-cut quartz crystal (diameter, 13.7 mm) with gold electrodes (diameter, 5.1 mm) on both sides as the working electrode (WE). The WE surface was cleaned by repeatedly applying potential from −0.5 V to +1.0 V in a 1 mol dm^−3^ NaClO_4_ solution just before use. A platinum wire and a silver–silver chloride (Ag/AgCl) electrode with 1 mol dm^−3^ LiCl were employed as the counter and reference electrodes, respectively. The reference electrode was connected to the sample solution through a ceramic frit as a liquid junction. In the EQCM measurements, the current and frequency of the WE were scanned at 298 K in an electronic thermostatic chamber (TB-1, BAS, Tokyo, Japan) by applying a predetermined potential after deaeration by bubbling nitrogen gas for 10 minutes. Nitrogen gas was flowed through the electrolytic cell during the measurements. The deposition weight change on the electrode surface, Δ*m*, was obtained from the frequency change of the AT-cut quartz crystal, Δ*f*, as shown in eqn (1),1
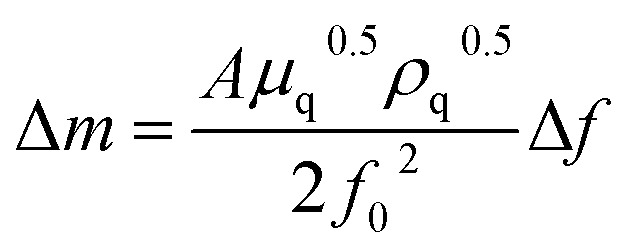
where *f*_0_, *μ*_q_, *ρ*_q_, and *A* represent the center frequency (*f*_0_ = 7.995 MHz), shear stress of quartz (*μ*_q_ = 2.947 × 10^11^ g cm^−1^ s^−2^), crystal density (*ρ*_q_ = 2.684 g cm^−3^), and electrode area (*A* = 0.196 cm^2^), respectively. The EQCM measurements were conducted in 1 mol dm^−3^ NaClO_4_ at pH 3–4 containing 1 mmol dm^−3^ U^VI^O_2_^2+^.

Electrochemical impedance spectroscopy (EIS) was carried out with a ModuLab XM ECS (Solartron Metrology, United Kingdom) using a three-electrode electrochemical cell (3.0 × 10^−3^ dm^3^) with a gold electrode (electrode diameter, 3 mm; electrode area, 0.07 cm^2^; BAS, Tokyo, Japan) and a coiled Pt wire (length, 23 cm; diameter of the coil, 0.5 mm) employed as the working and counter electrodes, respectively. The WE was polished with diamond and alumina abrasives immediately before use. The reference electrode was an Ag/AgCl wire in a reference solution (3 mol dm^−3^ NaCl). The reference electrode was connected to the sample solution through a porous glass frit as a liquid junction. EIS were measured as follows. The U deposits were formed on the WE surface by pre-electrolysis applying −0.35 V (*vs.* Ag/AgCl), at which the U^VI^ reduces to U^V^ in 1 mol dm^−3^ NaClO_4_ at pH 4 containing 1 mmol dm^−3^ U^VI^O_2_^2+^ after deaeration by bubbling nitrogen gas for 10 minutes. Then, the EIS of the WE covered by U deposits was measured at 298 K in an electronic thermostatic chamber with a potential of −0.35 V (*vs.* Ag/AgCl) in 1 mol dm^−3^ NaClO_4_ at pH 4. Analysis of the obtained spectra used an equivalent circuit of the electrode covered with U deposits in Zview software (Scribner Associates Inc., North Carolina, USA).

### X-ray absorption spectral measurements

X-ray absorption spectra at the U L3-edge were recorded in the 16.86–17.90 keV range using synchrotron radiation at beamline BL14B1, SPring-8. Incident photon energy was calibrated by Y K-edge (17.04 keV) in Y oxide. Extended X-ray absorption fine structure (EXAFS) spectra were analyzed (*k*-range, 0–13 Å^−1^; *k*-weight, *k*^2^) using Athena.^23^

## Results and discussion

### Deposition behaviors

**Fig. 1:**
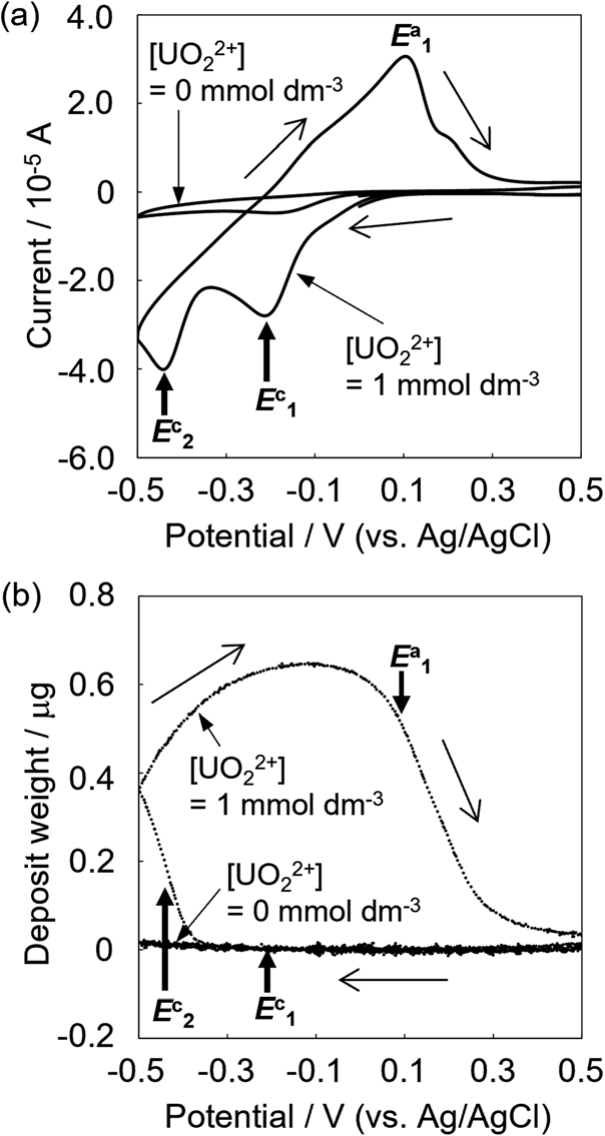
Current (a) and deposition (b) behaviors of uranyl ion (U^VI^O_2_^2+^) at pH 3.50 during cyclic voltametric measurements. Sample: [U^VI^O_2_^2+^] = 1 mmol dm^−3^, [NaClO_4_] = 1 mol dm^−3^, pH 3.50; scan rate 0.02 V s^−1^.

The deposition behaviors of uranyl ion (U^VI^O_2_^2+^) at pH 3.50 during cyclic voltametric measurements were investigated by EQCM. The voltammogram of U^VI^O_2_^2+^ exhibited two reduction peaks (E_1_^c^ and E_2_^c^ in Fig. 1a) observed at potentials around −0.2 and −0.4 V (*vs.* Ag/AgCl), respectively. In our previous study, the time courses of the reduction current and the increase in deposits following U^VI^O_2_^2+^ reduction by applying −0.35 V (*vs.* Ag/AgCl) were investigated.^13^ No deposition was observed immediately after the start of electrolysis, which was the reduction from U^VI^ to U^V^. Thus, E_1_^c^ indicates U^VI^ reduction to U^V^ and deposition was not detected at that potential (Fig. 1b). Deposition started at around −0.35 V (*vs.* Ag/AgCl). It has been reported that U deposits on the electrode surface accelerate the rate of the disproportionation of U^V^O_2_^+^ and promote the reduction of U^V^O_2_^+^ (catalytic effect by U deposits).^20^ Therefore, the E_2_^c^ behavior indicates the reduction of U^V^O_2_^2+^ to U^4+^ with a catalytic effect by U deposits. A decrease in deposits was observed with the increase in the oxidation current at around E_1_^a^. This decrease is due to the oxidation dissolution of U deposits.

**Fig. 2:**
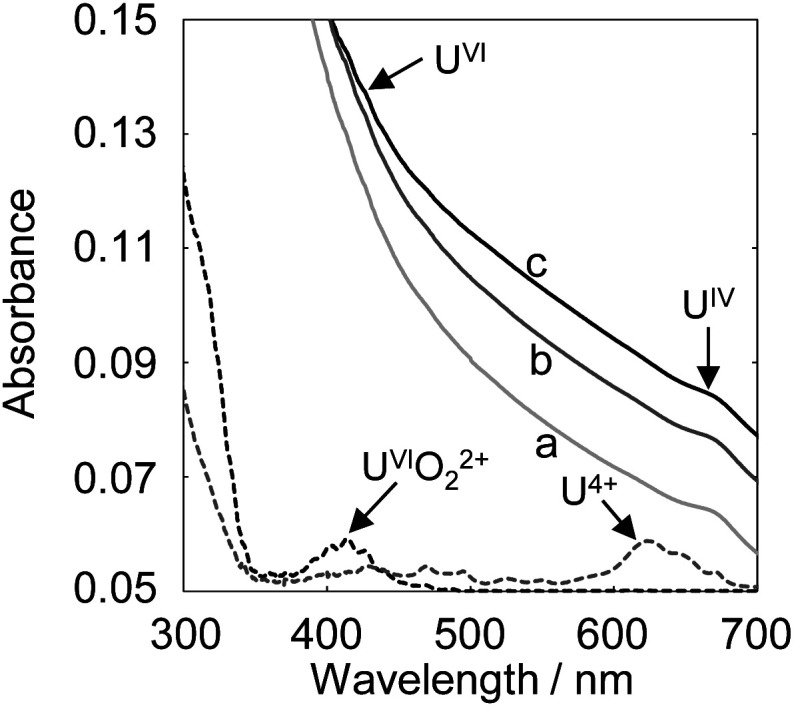
The change over time of the absorption spectrum of the U^V^ solution. Line a, 4 min; line b, 10 min; line c, 30 min; sample: [U^V^O_2_^+^] = 1 mmol dm^−3^, [NaClO_4_] = 1 mol dm^−3^, pH 3.50. Black dashed line, spectrum of U^VI^O_2_^2+^; grey dashed line, spectrum of U^4+^.

Since electrolytic-reduced monocationic U^V^O_2_^+^ has a smaller charge than dicationic U^VI^O_2_^2+^, it is less likely to be deposited. The absorption spectrum changes of the U^V^ solution over time were measured to confirm the oxidation state of the dissolved species that trigger deposition. The U^V^ solution was prepared by electrolysis from U^VI^ to U^V^ at −0.35 V (*vs.* Ag/AgCl) for 1870 s in a pH 2.8 solution as in the previous study.^20^ In the spectrum immediately after (Fig. 2, line a), the baseline rose due to the light scattering of U deposits and an absorption peak at around 650 nm attributed to U^IV^ was observed. Over time (Fig. 2, lines b and c), the light scattering by U deposits increased and U^VI^ absorption was detected at around 450 nm. These results indicate that U^IV^, with its high charge density and low solubility,^24^ was generated by the U^V^ disproportionation and U^IV^ was deposited.

### The chemical reaction of U deposits

**Fig. 3:**
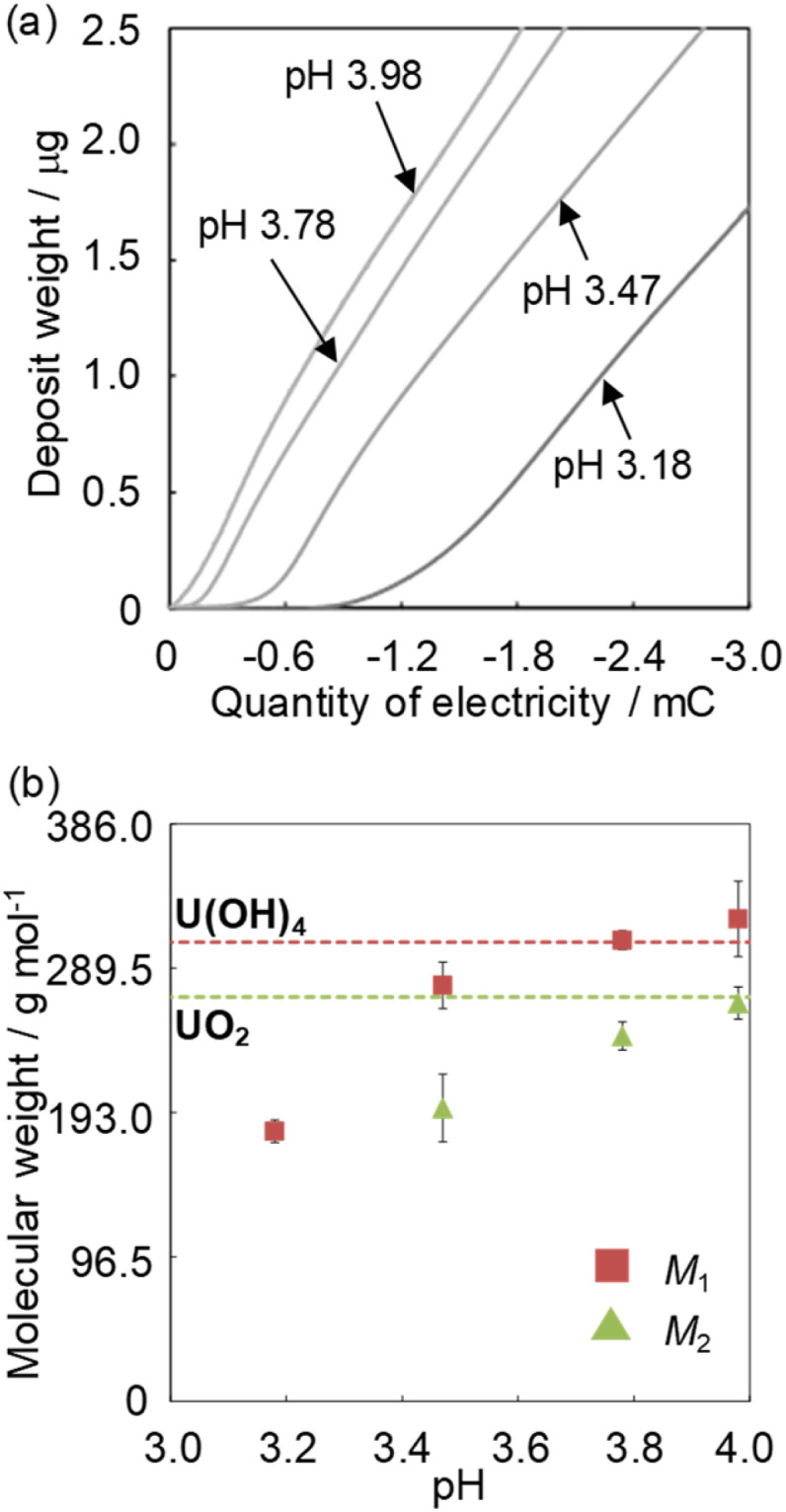
The relationship between the electricity quantity and the deposit weight (a) and the pH dependence of the obtained *M*_1_ and *M*_2_ (b). Sample: [U^VI^O_2_^+^] = 1 mmol dm^−3^, [NaClO_4_] = 1 mol dm^−3^.

The deposition increase by electrolytic U reduction was investigated by EQCM to estimate the species of deposits formed on the electrode. Fig. 3a shows the relationship between deposit weight (*M*_*n*_) and electricity quantity (*Q*) during electrolysis when applying −0.35 V (*vs.* Ag/AgCl) in solutions with various pH values. The *Q* values were obtained from the difference between the electricity quantities of the uranium sample and those of the blank sample. In the high pH solution, U begins to deposit at smaller electricity. The U deposition occurred soon after the start of electrolysis in the solution of around pH 4.

Assuming that all electrolysis products deposit, the molecular weight of such deposits can be obtained from the relationship between the electricity quantity and deposit amount (*m*) as2
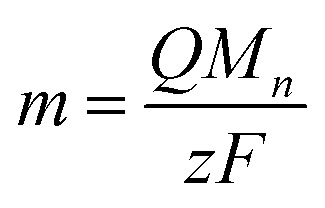
where *z* is the number of electrons contributed to the reduction, which is −2 because the deposition follows the reduction from U^VI^ to U^IV^, and *F* is Faraday's constant.

Though the variation of deposition weight with respect to the electricity quantity was linear at pH 3.18, two linear relations were observed above pH 3.47. The *M*_1_ values of the deposits immediately after deposition at pH 3.18–4.0 and the *M*_2_ values of the second linear relation at pH 3.47–4.0 were obtained from eqn (2) (Fig. 3b). The *M*_1_ obtained at pH 3.18 was *ca.* 190. It is unlikely that the molecular weight of the deposits would be smaller than the elemental weight of natural U (^238^U). In the Eh-pH diagram of U,^25^ U^IV^ exists as a hydroxide. Also, D. Rai *et al.* estimate the concentration of U^IV^(OH)_3_^+^(aq) in equilibrium with U^IV^O_2_·*x*H_2_O(am) at pH 3–3.5 to be about 10^−4.7^–10^−5.7^ mol dm^−3^ and the concentration of U^IV^(OH)_4_(aq) in equilibrium with U^IV^O_2_·*x*H_2_O(am) at around pH 4 to be about 10^−8^ mol dm^−3^.^24^ Since the solubility of U^IV^ hydroxide decreases significantly from pH 3 to 4, more reduction products are required for deposition at low pH and the diffusion of some of U^IV^ into the bulk solution phase cannot be ignored and is thought to have decreased the reduction/deposition efficiency. As a result, the apparent molecular weight decreased at pH < 3.8. The *M*_1_ obtained at pH 4 was 322 ± 25. This value is close to that of U^IV^ hydroxide (molecular weight of ^238^U^IV^(OH)_4_, 306). The *M*_2_ obtained at pH 4 was 266 ± 11. This value was close to that of U^IV^ dioxide (molecular weight of ^238^U^IV^O_2_, 270). D. Gil *et al.* obtained nanoscale U^IV^O_2_ by dropping a reduced U^IV^ solution into an alkaline bath at pH 4.5–5.^26^ The obtained result agreed with the chemical state of U deposits in the previous study. M. C. Rath *et al.* reported that the formation of U^IV^O_2_ nanoparticles occurred in pH 2.5–3.7 aqueous solutions containing 10 mmol dm^−3^ uranyl nitrate and 10% 2-propanol with an irradiating electron beam.^27^ The amount of U^IV^O_2_ formed increases with higher pH, which is similar to the formation behavior of U^IV^O_2_ in this study. This could be due to differences in the nucleation and growth processes at each pH condition. These results indicate the following: for the U deposit formation, it is considered that U^IV^ ions are formed by disproportionation of U^V^ deposited as U^IV^ hydroxide. U^IV^ hydroxide does not change at pH < 3.2 and the chemical state changes from the hydroxide to the oxide at pH > 3.5.

**Fig. 4:**
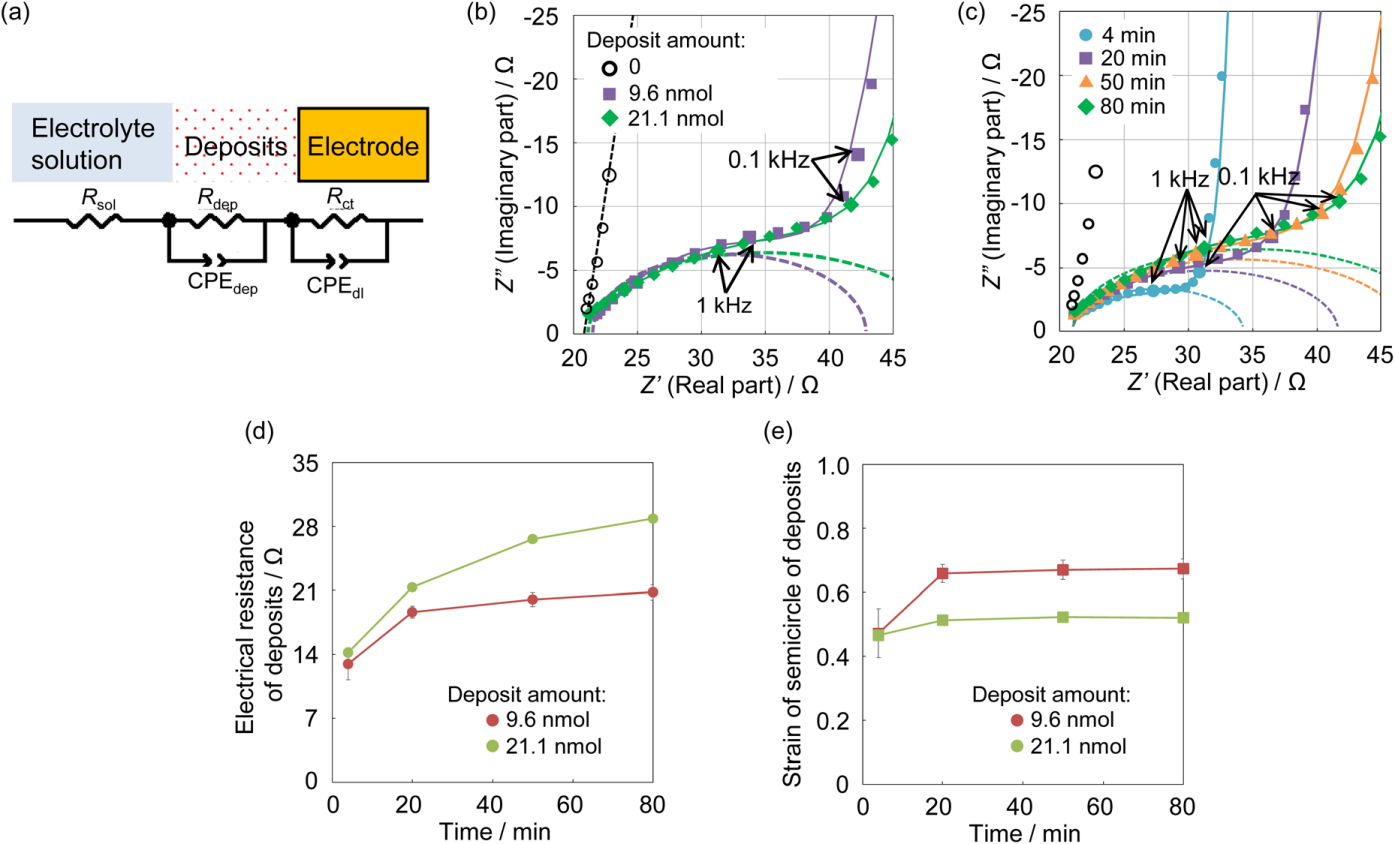
The equivalent circuit of the electrode covered by U deposits (a). *R*_sol_, electrical resistance of solvent; *R*_dep_, electrical resistance of deposits; *R*_ct_, charge transfer resistance; CPE_dep_, the constant phase element of deposits; CPE_dl_, the constant phase element of the electrode. Impedance spectra of U deposits by the deposit weight (b) and 4 to 80 minutes after the cessation of forming deposits (c). Sample, [NaClO_4_] = 1 mol dm^−3^, pH 4.0. The solid lines are fittings of the equivalent circuit. The dashed lines are the semicircle of deposits. Electrical resistance (d) and strain of the semicircle (e) of U deposits.

The impedance spectra of U deposits formed on the electrode surface at pH 4.0, the condition at which the chemical state of the U deposits changed, were measured to clarify the electrochemical parameters of the U deposits. Analysis of the obtained spectra used an equivalent circuit of the electrode covered with U deposits. This was assumed to be a circuit with the electrical resistance (*R*_dep_) of the deposited film formed in a solution at pH 4, its electrical capacitance (*C*_dep_), charge transfer resistance (*R*_ct_), electric double-layer capacitance (*C*_dl_), and solution resistance (*R*_sol_).^28^ The capacitance element was used as a constant phase element (CPE)^28^ because the capacitive semicircle deviated from a perfect circle (Fig. 4a). The impedance spectrum of the equivalent circuit containing CPE is shown in eqn (3),3
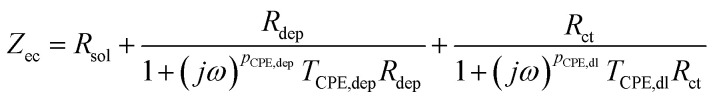
where *p*_CPE,dep_ is the strain of the semicircle of the deposits, *T*_CPE,dep_ is a value related to the electrical capacitance, *p*_CPE,dl_ is the strain of the semicircle of the electrode, and *T*_CPE,dl_ is a value related to the electrical capacitance.

The obtained impedance spectrum was observed in the change derived from U deposits on the electrode surface (Fig. 4b). The spectrum agreed well with the fitting from eqn (3). The obtained electrochemical parameters of the U deposits are shown in Tables S1 and S2 (ESI†). In the impedance spectra of the 9 and 21 nmol U deposits, the capacitive semicircle diameter increased as the deposit weight increased. The change on the electrode surface from U deposit weight was observed in the spectrum. The spectra were measured at 4 to 80 minutes after the cessation of deposit formation (Fig. 4c). The obtained electrical resistances of the U deposits were around 14 Ω at 4 minutes after the end of electrodeposition and increased with standing time (Fig. 4d). This indicates that the U deposits changed to a compound with higher electrical resistance. The obtained strain values of the semicircle of U deposits were around 0.5 at 4 minutes after stopping electrodeposition and increased with time (Fig. 4e). These values indicate the heterogeneity of the deposits and suggest that the U deposits change from an amorphous compound to a crystalline compound. Therefore, it is considered that the U deposits change from the U^IV^ hydroxide, a low electrical resistance amorphous compound, to the U oxide, which is generally a crystalline compound with higher electrical resistance than a hydroxide.

### Identification of U deposits

**Fig. 5:**
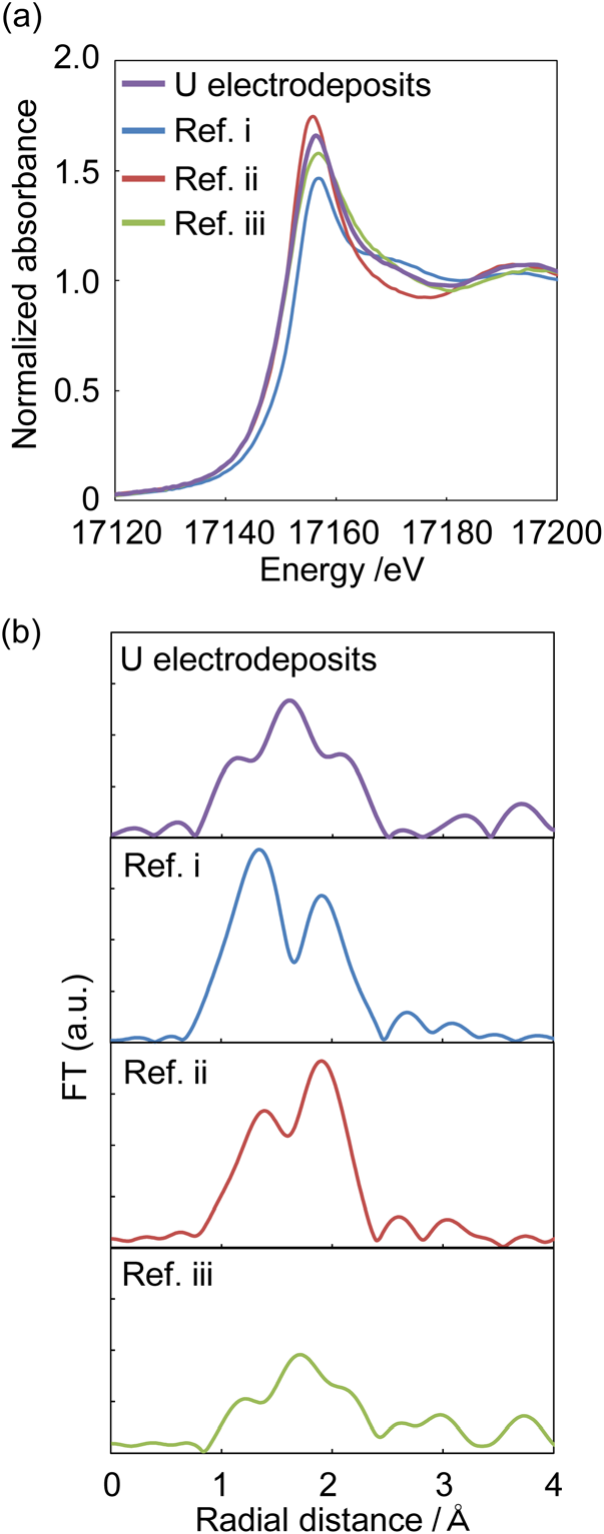
The XANES (a) and EXAFS (b) of U electrodeposits and reference samples. Sample: [U^VI^O_2_^+^] = 10 mmol dm^−3^, [NaClO_4_] = 1 mol dm^−3^, pH 2; Ref. i: [U^VI^O_2_^+^] = 10 mmol dm^−3^, [HClO_4_] = 0.2 mol dm^−3^, [NaClO_4_] = 0.8 mol dm^−3^; Ref. ii: [U^IV^/U^VI^] = 10 mmol dm^−3^, [HClO_4_] = 0.2 mol dm^−3^, [NaClO_4_] = 0.8 mol dm^−3^; Ref. iii: [U^IV^/U^VI^] = 10 mmol dm^−3^, [NaClO_4_] = 1 mol dm^−3^, pH 4.

XAFS spectra were measured to identify the starting U deposits. U deposits were prepared at 298 K in an electronic thermostatic chamber with a potential of −0.35 V (*vs.* Ag/AgCl) after deaeration by bubbling nitrogen gas for 10 minutes in 1 mol dm^−3^ NaClO_4_ at pH 2, and the U chemical state was not changed. Nitrogen gas was flowed through the electrolytic cell during the measurements. The U deposits were transferred from the electrolytic cell to a quartz cell in a nitrogen gas-displaced glove bag and placed in a gas-barrier zipper bag together with an oxygen scavenger. As reference samples, (i) U^VI^O_2_^2+^ in 0.2 mol dm^−3^ HClO_4_ solution was prepared to contribute hexavalent U and uranyl oxygen; (ii) U^4+^ was prepared by electrolytic reduction of Ref. i for tetravalent U and oxygen of coordinated water; and (iii) U^IV^ hydroxide was prepared by neutralizing Ref. ii with NaOH to contribute tetravalent U and the oxygen of the hydroxyl group. As shown in Fig. 5a, the X-ray absorption near edge structure (XANES) spectra of Ref. i–iii indicate that the absorption near the edge of the tetravalent ion was observed at a lower energy than that near the hexavalent ion. This result was similar to that of Pierce's study.^29^ The XANES of the U electrodeposits were similar to those of the U^4+^ and U^IV^ hydroxide samples. Therefore, the U electrodeposit oxidation state was tetravalent. In the EXAFS spectrum of the U deposits, oxygen was observed at 1.2, 1.5, and 2.1 Å (Fig. 5b). For Ref. i (U^VI^O_2_^2+^ in 0.2 mol dm^−3^ HClO_4_), uranyl oxygen and the oxygen of coordinated water were observed at 1.3 and 1.9 Å, respectively (Fig. 5b, Ref. i). The peak positions of the EXAFS spectra appear at a shorter distance than the real interatomic distance due to the phase shift effect. This result was similar to that of Thompson's previous study.^30^ Ref. ii (U^4+^ in 0.2 mol dm^−3^ HClO_4_) also showed uranyl oxygen and the oxygen of coordinated water. The binding distance of the coordinated water oxygen was similar to that of Th^4+^.^31^ It is considered that uranyl oxygen was detected because some U^4+^ was reoxidized to U^VI^O_2_^2+^ over the several days between the preparation of the U^4+^ sample and the XAFS measurement. Since the peak height of the oxygen of coordinated water was higher than that of uranyl oxygen, U^IV^ was the major valence state. The EXAFS of Ref. iii (U^IV^ hydroxide, pH 4.0) showed the oxygen of the hydroxyl group at 1.5 Å. Since the U electrodeposits showed the oxygen of the hydroxyl group, the electrodeposits can be identified as U^IV^ hydroxide. The above results clarified that U^IV^ ions formed deposits as U^IV^ hydroxide.

## Conclusions

**Fig. 6:**
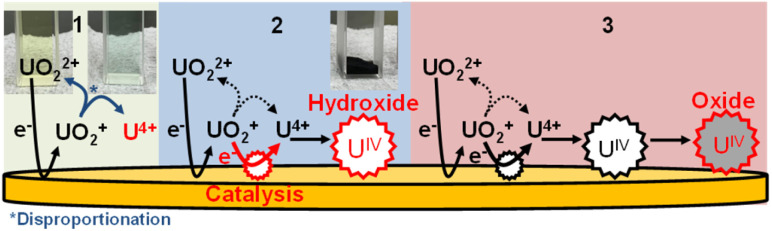
The proposed mechanism of U electrodeposition.

From these results, we propose the following deposition mechanism (Fig. 6): (1) U^IV^ is formed by U^V^ disproportionation following electrolytic U^VI^ reduction. (2) U^IV^ hydroxide is formed as a compound immediately after deposition. (3) U^IV^ hydroxide finally changes to U^IV^ oxide, with a higher electrical resistance than the hydroxide. Note that this is the first report explaining the deposition mechanism following U reduction.

## Author contributions

K. Ouchi and Y. Kitatsuji conceived the project and acquired funding. K. Ouchi conducted UV-vis absorption spectroscopy and electrochemical measurements and curated these data. D. Matsumura and T. Tsuji measured XAFS spectra. All authors contributed to the discussion of the results.

## Conflicts of interest

There are no conflicts to declare.

## Supplementary Material

RA-013-D3RA02899B-s001
