# Supplementary material for: Uranium hydroxide/oxide deposits on uranyl reduction
Source: RSC Adv. 2023 May 31;13(24):16321–6. doi: 10.1039/d3ra02899b (PMC10230268; doi:10.1039/d3ra02899b)
Supplement: RA-013-D3RA02899B-s001 [file RA-013-D3RA02899B-s001.pdf]

Electronic Supplementary Material (ESI) for RSC Advances.
This journal is © The Royal Society of Chemistry 2023

Supplementary Information

Uranium Hydroxide/Oxide Deposits on Uranyl Reduction

Kazuki Ouchi,* Daiju Matsumura, Takuya Tsuji, Tohru Kobayashi, Haruyoshi Otobe and Yoshihiro Kitatsuji

Abstract: We clarified the chemical reaction of deposits following the reduction of uranyl ion ($U^{VI}O_2^{2+}$) from the results of electrochemical quartz crystal microbalance, impedance spectra and X-ray absorption fine structure measurements. We propose the following deposition mechanism: 1) U^{IV} is formed by the disproportionation of U^{V}. 2) U^{IV} forms U^{IV} hydroxide deposits, and 3) finally, the hydroxide deposits change to U^{IV} oxide, generally having a larger electrical resistance than the former.

Table of Contents

Results 3

Table S1 The results of fitting by the equivalent circuit of the electrode covered by 9.6 nmol of U deposits. 3

Table S2 The results of fitting by the equivalent circuit of the electrode covered by 21.1 nmol of U deposits. 3

Results

Table S1 The results of fitting by the equivalent circuit of the electrode covered by 9.6 nmol of U deposits.

	4 min	20 min	50 min	80 min
R_{sol} /Ω	(1.97 ± 0.08)×10	(2.11 ± 0.03)×10	(2.12 ± 0.03)×10	(2.11 ± 0.03)×10
R_{dep} /Ω	(1.3 ± 0.2)×10	(1.86 ± 0.07)×10	(2.00 ± 0.08)×10	(2.08 ± 0.08)×10
$p_{CPE,dep}$	0.47 ± 0.08	0.66 ± 0.03	0.67 ± 0.03	0.67 ± 0.03
$T_{CPE,dep}$ /Fs^{p-1}	(10 ± 6)×10^{-4}	(1.3 ± 0.3)×10^{-4}	(1.0 ± 0.3)×10^{-4}	(9 ± 3)×10^{-5}
R_{ct} /Ω	(7.6 ± 0.3)×10^{4}	(1.03 ± 0.03)×10^{5}	(1.9 ± 0.1)×10^{5}	(2.05 ± 0.01)×10^{5}
$p_{CPE,dl}$	0.993 ± 0.003	0.939 ± 0.002	0.928 ± 0.002	0.924 ± 0.002
$T_{CPE,dl}$ /Fs^{p-1}	(2.54 ± 0.02)×10^{-4}	(2.26 ± 0.01)×10^{-4}	(2.16 ± 0.01)×10^{-4}	(2.12 ± 0.01)×10^{-4}

Table S2 The results of fitting by the equivalent circuit of the electrode covered by 21.1 nmol of U deposits.

	4 min	20 min	50 min	80 min
R_{sol} /Ω	(1.97 ± 0.02)×10	(1.99 ± 0.09)×10	(1.98 ± 0.01)×10	(1.98 ± 0.01)×10
R_{dep} /Ω	(1.42 ± 0.04)×10	(2.13 ± 0.02)×10	(2.66 ± 0.04)×10	(2.88 ± 0.04)×10
$p_{CPE,dep}$	0.47 ± 0.02	0.514 ± 0.007	0.5235 ± 0.008	0.5210 ± 0.008
$T_{CPE,dep}$ /Fs^{p-1}	(1.1 ± 0.2)×10^{-3}	(6.3 ± 0.4)×10^{-4}	(5.1 ± 0.4)×10^{-4}	(5.1 ± 0.3)×10^{-4}
R_{ct} /Ω	(1.5 ± 0.5)×10^{6}	(1.23 ± 0.02)×10^{5}	(1.22 ± 0.03)×10^{5}	(1.18 ± 0.02)×10^{5}
$p_{CPE,dl}$	0.9895 ± 0.0009	0.9659 ± 0.0008	0.9498 ± 0.0008	0.9468 ± 0.0008
$T_{CPE,dl}$ /Fs^{p-1}	(5.70 ± 0.01)×10^{-4}	(4.930 ± 0.006)×10^{-4}	(4.737 ± 0.008)×10^{-4}	(4.594 ± 0.008)×10^{-4}
